# Development of a healthy ageing index in Latin American countries - a 10/66 dementia research group population-based study

**DOI:** 10.1186/s12874-019-0849-y

**Published:** 2019-12-05

**Authors:** Christina Daskalopoulou, Kia-Chong Chua, Artemis Koukounari, Francisco Félix Caballero, Martin Prince, A. Matthew Prina

**Affiliations:** 1 Department of Health Service and Population Research, King’s College London, Institute of Psychiatry, Psychology and Neuroscience, De Crespigny Park, London, SE5 8AF UK; 2 Department of Infectious Disease Epidemiology, London School of Hygiene & Tropical Medicine, Faculty of Epidemiology and Population Health, London, WC1E 7HT UK; 3 Department of Psychiatry, Universidad Autónoma de Madrid, 4 Arzobispo Morcillo, 28029 Madrid, Spain; 4 CIBER of Mental Health, Madrid, Spain; 5 Hospital Universitario de La Princesa, Instituto de Investigación Sanitaria Princesa (IP), Madrid, Spain

**Keywords:** 10/66, Healthy ageing metric, Psychometric properties, Measurement invariance, Bifactor model

## Abstract

**Background:**

Our population is ageing and in 2050 more than one out of five people will be 60 years or older; 80% of whom will be living in a low-and-middle income country. Living longer does not entail living healthier; however, there is not a widely accepted measure of healthy ageing hampering policy and research. The World Health Organization defines healthy ageing as the process of developing and maintaining functional ability that will enable well-being in older age. We aimed to create a healthy ageing index (HAI) in a subset of six low-and-middle income countries, part of the 10/66 study, by using items of functional ability and intrinsic capacity.

**Methods:**

The study sample included residents 65-years old and over (*n* = 12,865) from catchment area sites in Cuba, Dominican Republic, Peru, Venezuela, Mexico and Puerto Rico. Items were collected by interviewing participants or key informants between 2003 and 2010. Two-stage factor analysis was employed and we compared one-factor, second-order and bifactor models. The psychometric properties of the index, including reliability, replicability, unidimensionality and concurrent convergent validity as well as measurement invariance per ethnic group and gender were further examined in the best fit model.

**Results:**

The bifactor model displayed superior model fit statistics supporting that a general factor underlies the various items but other subdomain factors are also needed. The HAI indicated excellent reliability (*ω* = 0.96, *ω*_*Η*_ = 0.84), replicability (*H* = 0.96), some support for unidimensionality (*Explained Common Variance* = 0.65) and some concurrent convergent validity with self-rated health. Scalar measurement invariance per ethnic group and gender was supported.

**Conclusions:**

A HAI with excellent psychometric properties was created by using items of functional ability and intrinsic capacity in a subset of six low-and-middle income countries. Further research is needed to explore sub-population differences and to validate this index to other cultural settings.

**Electronic supplementary material:**

The online version of this article (10.1186/s12874-019-0849-y) contains supplementary material, which is available to authorized users.

## Background

Globally, life expectancy has increased by an average of 5 years between 2000 and 2015 [1]. The number of people aged 60 and over is expected to double by 2050 and Latin American countries are to experience the fastest growth over the next 15 years [2]. Risk of disability and noncommunicable chronic diseases increases with age as well [3]. Despite technological and medical advances people are likely to experience multimorbidity in later life, living with multiple chronic conditions [3]. This growing population of frail older people will lead to higher health and societal costs. There is, therefore, an imperative need to examine the ageing process and most importantly the elements that will enable people to live longer and in a healthy way.

Systematic reviews indicate that till now there is neither a unanimous definition nor a standardised metric of healthy ageing and the percentage of healthy or successful agers differs considerably among studies mainly due to lack of common definition and measurement procedures [4, 5]. However, the need for an aggregated metric of health status that would permit valid comparisons among populations and over time is well recognised [6, 7]. Constant monitoring of the health status of older people will enable us to identify key determinants and implement comprehensive healthy ageing policies. According to the latest report of ageing and health from the World Health Organization (WHO), healthy ageing is defined as the process of developing and maintaining the functional ability that will enable well-being in older people [3].

In this study, our key objective is the creation of a healthy ageing index (HAI) based on the WHO conceptual framework in a subset of Latin American countries. We also aim to examine this index for various psychometric properties (i.e. omega reliability coefficients, explained common variance measure of unidimensionality, index *H* of construct replicability and concurrent convergent validity) and measurement invariance for ethnic groups and gender.

## Methods

### 10/66 study

Data were collected from specific urban and rural catchment areas in Latin America (Cuba, Dominican Republic, Peru, Venezuela, Mexico and Puerto Rico); part of the 10/66 Dementia Research Group (10/66 DRG) survey. 10/66 DRG is a multicentre study on ageing and dementia performed in low-and-middle income countries. Baseline face-to-face interviews of residents 65 years old and over were carried out between 2003 and 2007 in all areas, other than Puerto Rico where baseline data were collected between 2007 and 2010. Catchment areas were selected to be broadly representative of the source community and 2000 target participants per country (with the exemption of 3000 in Cuba) were chosen a priori; response rate was excellent ranging from 80 to 95%. Participant and informant interviews as well as physical examination were part of the 10/66 study protocol. In cases where the participant’s capacity to provide reliable information was in doubt, the information was corroborated by an informant (usually a relative or a caregiver). A more detailed description is available at www.alz.co.uk/1066 and elsewhere [8, 9].

### Healthy ageing indicators

Questions measuring health and disability according to the International Classification of Functioning, Disability and Health (ICF) [10] were used as healthy ageing indicators/items to build the index. A total of 26 health questions, which were either self-reported by the participants or provided by key informants, were identified from various questionnaires and operationalised as described below. Difficulties with: household responsibilities, walking a kilometre, washing whole body, getting dressed, carrying out work and everyday activities, making decisions, using the toilet, handling money, finding the right word, completing chores, routine (assessed as: ‘feeling of not coping properly with everyday routine’), sleep (assessed as: ‘trouble with sleep or recent change in pattern’), orientation (assessed as: ‘forgets where he/she is’); hearing and eye problems, change in daily activities, exhaustion (assessed as: ‘gets worn out or exhausted during daytime or evening’) and speed test (assessed by the time in seconds taken to walk 10 m). Finally, cognitive assessment included the following items: instant recall (a 10 word list learning assessed for three times; we considered one value: the maximum number of words among the three trials), delayed recall (assessed as: ‘do you remember the three words I told you a few minutes ago’), long term memory item (correctly remembering the name of a well-known person linked to a historical event), immediate recall (assessed by repeating three words that previously were mentioned by the interviewer), verbal fluency (assessed by the number of animals that the participant could recall in 1 minute), time orientation (day, month, year, season), story recall (repeat a story that just heard from the interviewer), and praxis (fold a piece of paper, following instructions). Most items were categorical and in some cases where a continuous outcome was reported (i.e. instant recall, verbal fluency, speed test, story recall), we divided the whole sample in three groups according to the lower and upper quartiles of each distribution; values below the 25th percentile, between the 25th and 75th and above the 75th indicated high, moderate and low performance, respectively. Higher values indicated worse health outcomes. Table 1 provides more details on items origin (i.e. participant or informant interview; initial questionnaire from the 10/66 interview from which they were extracted).

**Table 1:** Healthy Ageing Indicators Origin

Items/Indicators	Origin	Questionnaire
Household responsibilities difficulty	Participant	WHO-DAS II
Walking a km difficulty	Participant	WHO-DAS II
Washing whole body difficulty	Participant	WHO-DAS II
Getting dressed difficulty	Participant	WHO-DAS II
Carrying out work & everyday activities difficulty	Participant	WHO-DAS II
Making decisions difficulty	Informant	CSI’D’-RELSCORE
Using the toilet difficulty	Informant	CSI’D’-RELSCORE
Handling money difficulty	Informant	CSI’D’-RELSCORE
Hearing problem	Participant & informant	Health (including pain and impairments)
Eye problem	Participant & informant	Health (including pain and impairments)
Finding right word difficulty	Informant	CSI’D’-RELSCORE
Change in daily activities	Informant	CSI’D’-RELSCORE
Forgets where he/she is	Informant	CSI’D’-RELSCORE
Difficulty completing chores	Informant	CSI’D’-RELSCORE
Sleep trouble or recent change in pattern	Participant	Mental Health (GMS-version B3)
Feeling of not coping properly with everyday routine	Participant	Mental Health (GMS-version B3)
Gets worn out or exhausted during daytime or evening	Participant	Mental Health (GMS-version B3)
Time in seconds taken to walk 10 m	Clinical examination	neurological assessment (NEUROEX)
Learn test	Participant	10 word list learning
Delayed recall	Participant	CSI’D’
Long memory test	Participant	CSI’D’
Immediate recall	Participant	CSI’D’
Verbal fluency	Participant	CSI’D’
Time orientation	Participant	CSI’D’
Praxis-fold a piece of paper	Participant	CSI’D’
Story recall difficulty	Participant	CSI’D’

*WHO-DAS II* World Health Organization. Disabilty Assessment Schedule 2.0; *CSI’D’-RELSCORE* Community Screening Interview for Dementia-Informant Scale, *GMS* Geriatric Mental State Interview, *NEUROEX* Neurological Examination, *CSI’D’* Community Screening Interview for Dementia

### Data analyses

We used SPSS version 22 for data management and Mplus 7.4 software for any statistical analyses. Mean and variance-adjusted weighted least-squares (WLSMV) estimator, suitable for the analysis of categorical data, polychoric correlations and theta parameterisation -in which residual variances of observed categorical outcome variables are allowed to be parameters in the models- were employed [11]. A pairwise present approach to missing data was used as it is the default in Mplus with WLSMV estimator [12].

Model accuracy was routinely reported by chi-square value with degrees of freedom (df); however, given the sensitivity of chi-square to large sample sizes, we used goodness-of-fit indices to make decisions about the global fit of the models and we inspected discrepancies between predicted and observed correlations to assess local fit [13]. We reported the comparative fit index (CFI) and root mean square error of approximation (RMSEA) with 90% confidence intervals (CI). We considered a model to have an acceptable fit when CFI ≥ 0.90 and RMSEA values close or less than 0.06 [14]. Nested models were compared by using the DIFFTEST command of MPLUS (an adjusted chi-square test when the WLSMV estimator is employed) [11, 13].

### Exploratory factor analysis

To identify the appropriate number of factors and the pattern of relationships between items and factors the data-driven methodology of exploratory factor analysis (EFA) was employed [15]. For the EFA, a 30% stratified by gender and country random sample of our initial sample was used and the remaining 70% was used as our validation sample in the confirmatory factor analyses (CFA). Parsimax (oblique) rotation, allowing for factor correlation and for minimum variable complexity, was employed to foster factor interpretability [16]. Factor loadings equal or higher than 0.20, in absolute value term, were considered as factor loading cut-off point. Response categories with less than 4% of the sample were merged with the adjacent higher category to avoid computational issues in the model fitting.

### Confirmatory factor analysis

As our objective was to build an index which would represent the multifaceted concept of healthy ageing, CFA framework was employed to identify the best measurement model representing healthy ageing as a single general construct. A second-order model and a bifactor model were considered, recognising the multidimensionality of healthy ageing but also focusing on an overall target construct; a one-factor model was also examined. Multidimensional measurement models without a general factor (i.e. first-order correlated-factors model) were not considered.

**Fig. 1:**
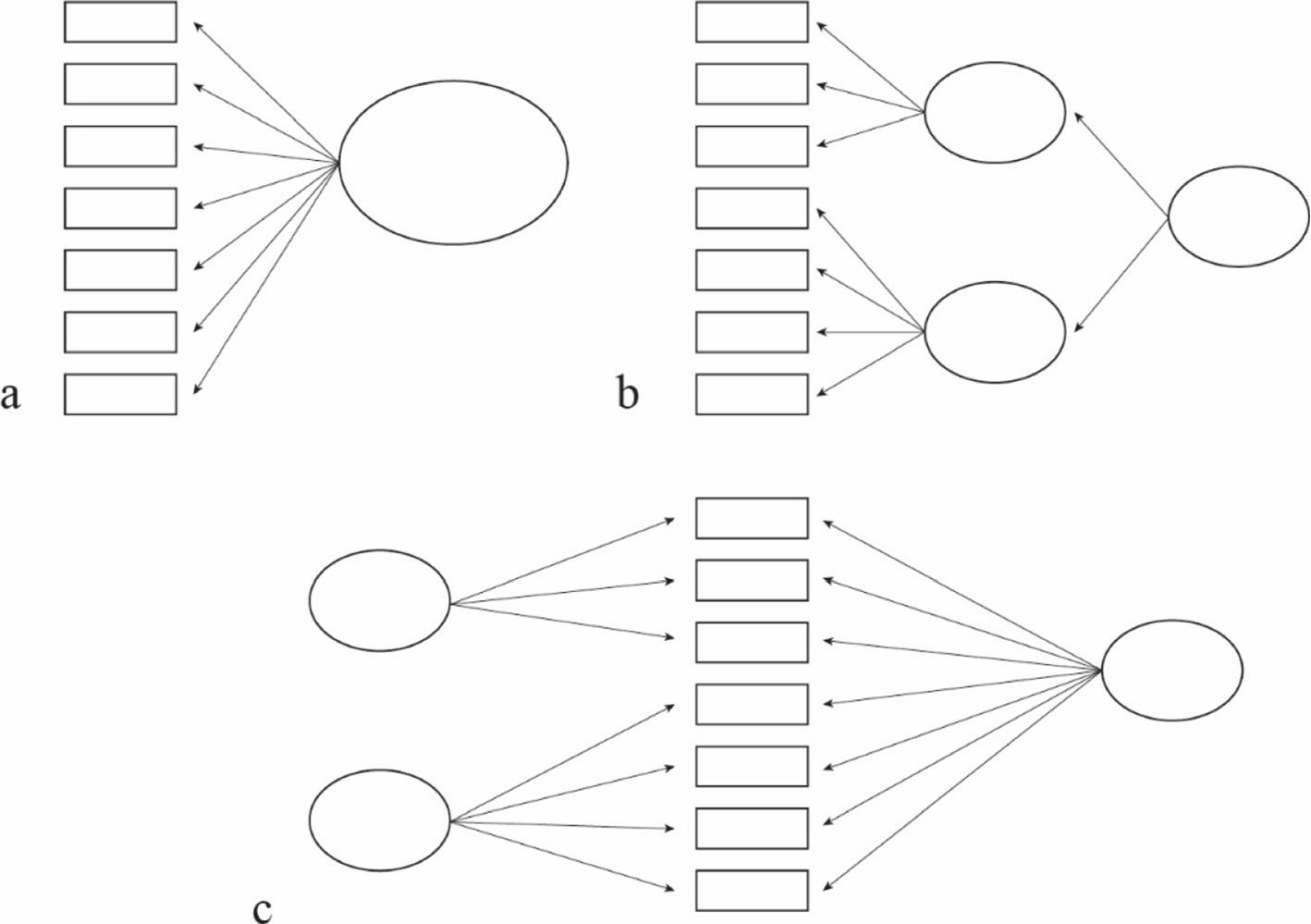
Graphical representation of a. one factor model; b. second-order; c. bifactor model

Figure 1 shows a simplified example of the structural difference among a one-factor, a second-order and a bifactor model. The main difference between a bifactor structure and a second-order model is that in the bifactor structure the general factor explains the covariance among all observed items, and not within first-order factors as is the case of the second-order model. Furthermore, a bifactor structure simultaneously allows to have other subdomain factors, which account for the variability not explained by the general factor [17].

Both the second-order and the bifactor model were set up by using the number and the item structure onto first-order and subdomain factors as suggested by the EFA. Hence, the second-order model was constituted by a second-order factor onto which the first-order factors of the EFA were loaded; the bifactor model was constituted by a general factor onto which all items were loaded and a number of orthogonal subdomain factors onto which items were loaded as suggested by the EFA. As a one-factor model and a second-order model are nested within the bifactor model the DIFFTEST command was used for model comparison [11, 18, 19].

### Measurement invariance

Since our data were collected from different populations, it was crucial to establish that our latent construct measured the same thing and in the same way across the different ethnic groups and across men and women. We assessed measurement invariance among the six countries and between men and women by performing multi-group confirmatory factor analyses (MGCFA) and creating nested models with increasing parameter constraints [20]. Measurement invariance within the factor analytic framework can be tested by examining the statistical fit of models that differ in the parameters that have been set to equal or not across groups [21]. A lack of invariance could indicate differences in the way a group interprets and replies to a measure. Measurement invariance was assessed in the model that was selected as superior in the fit by the confirmatory factor analyses. The analysis was carried out in three steps:

1. We checked if our model structure was the same across the different groups, meaning that our suggested model fitted the data well in all six countries and men and women separately.
2. We assessed configural invariance; a baseline multiple-group model was created in which all factor loadings and thresholds were freely estimated across groups (for identification purposes we set one item loading per factor fixed to 1 -referent indicator-, one threshold per item and one additional threshold for the referent indicator equal across groups, factor means fixed to zero to all groups and residual variances to one to the reference group) [21, 22]. This model constituted our baseline model for subsequent tests.
3. We assessed scalar invariance; all factor loadings and thresholds were constrained to equality across groups (for identification purposes factor means were fixed to zero and residual variances were fixed to one in the first group only). We did not assess metric invariance since in the invariance testing of ordinal items, loadings and thresholds cannot be tested separately [23]. (See Additional file 1 for MPLUS code).

To compare the models, the DIFFTEST command was used; however, the dependence of chi-square statistic on sample size makes it also a non-appropriate indicator for decrement fit evaluation between nested models [13]. For this reason we also examined the change in CFI (ΔCFI) and in the RMSEA (ΔRMSEA) goodness-of-fit. A change in CFI values less than or equal to 0.010 supplemented by a change of less than or equal to 0.015 in RMSEA would provide evidence for not rejecting the hypothesis of measurement invariance [19, 24].

### Psychometric coefficients

Omega (*ω*) and omega hierarchical (*ω*_*H*_) coefficients were calculated as they provide better estimates of measurement precision (reliability) than the traditional Cronbach’s alpha [25]. Omega coefficients estimate the proportion of variance in unit-weighted total score attributable to all sources of common variance and to the general factor within the bifactor framework [26–28]. A high *ω* value indicates a highly reliable multidimensional composite and a high *ω*_*H*_ value (> 0.80) in the bifactor structure indicates that the general factor is the dominant source of systematic variance with subdomain factors having less influence. We also calculated coefficient omega hierarchical subscales (*ω*_*ΗS*_) to estimate the strength of influence of subdomain factors. Coefficient *ω*_*ΗS*_ represents the proportion of reliable systematic variance of a subscale score after partitioning out general factor variability [29]. We also judged the unidimensionality of the index by calculating the Explained Common Variance index (*ECV*) [17, 30]. Higher values of *ECV* indicate a strong general factor allowing us to fit a unidimensional model even to multidimensional data. Finally, we checked for construct replicability (i.e. how well a set of items represents a latent variable) with the index *H*, which provides the proportion of variability in a latent construct explainable by its own indicators [31]. High values of *H* (> 0.80) indicate a well-defined latent variable which can be considered stable across settings. This index is of high importance in the structural equation model (SEM) framework as it assists in understanding the feasibility of a measurement model [28].

### Concurrent convergent validity

To examine the concurrent convergent validity of our index we estimated its association with the self-rated health (SRH) of the participants in the past 30 days. Multiple-indicators multiple-causes model (MIMIC) with latent variables was employed to eliminate any measurement unreliability from our conclusion [32]. Even though the SRH measure is quite subjective, since it is based on individuals’ opinion about their health status, research shows that it has strong predictive validity for mortality in general [33] and in the 10/66 cohort [34]. As a consequence, we estimated the associations of SRH with the healthy ageing latent construct, adjusted for age and sex, in the best fit measurement model.

## Results

### Sample study characteristics

Descriptive statistics of the study population are provided in Table 2.

**Table 2:** Characteristics of the 10/66 Cohort

Country	Total (%)	Cuba (%)	Dominican Republic (%)	Peru (%)	Venezuela (%)	Mexico (%)	Puerto Rico (%)
Total	12,865	2944	2011	1933	1965	2003	2009
Women	8288 (64%)	1913 (65%)	1325 (66%)	1183 (61%)	1252 (64%)	1268 (63%)	1347 (67%)
Men	4568 (36%)	1031 (35%)	684 (34%)	750 (39%)	713 (36%)	735 (37%)	655 (33%)
Age (years)							
65–69	3644 (28%)	760 (26%)	533 (27%)	554 (29%)	839 (43%)	544 (27%)	414 (21%)
70–74	3308 (26%)	789 (27%)	520 (26%)	493 (26%)	469 (24%)	581 (29%)	456 (23%)
75–79	2689 (21%)	639 (22%)	397 (20%)	399 (21%)	345 (18%)	426 (21%)	483 (24%)
80+	3211 (25%)	749 (25%)	561 (28%)	486 (25%)	308 (16%)	451 (23%)	656 (33%)
Marital Status							
Never married	1044 (8%)	275 (9%)	139 (7%)	213 (11%)	189 (10%)	105 (5%)	123 (6%)
Married/ cohabiting	5845 (45%)	1271 (43%)	586 (29%)	1092 (56%)	921 (47%)	1008 (50%)	967 (48%)
Widowed	4245 (33%)	928 (32%)	806 (40%)	524 (27%)	549 (28%)	766 (38%)	672 (33%)
Divorced/ separated	1644 (13%)	462 (16%)	465 (23%)	93 (5%)	261 (13%)	123 (6%)	240 (12%)
Education							
None	1370 (11%)	75 (3%)	392 (19%)	121 (6%)	156 (8%)	554 (28%)	72 (4%)
Some, did not complete primary	3606 (28%)	655 (22%)	1022 (51%)	231 (12%)	445 (23%)	864 (43%)	389 (19%)
Completed primary	3807 (30%)	979 (33%)	370 (18%)	727 (38%)	965 (49%)	351 (18%)	415 (21%)
Completed secondary	2483 (19%)	728 (25%)	135 (7%)	517 (27%)	266 (14%)	124 (6%)	713 (35%)
Tertiary (college)	1504 (12%)	499 (17%)	73 (4%)	321 (17%)	93 (5%)	108 (5%)	410 (20%)
Self-rated health in past 30 days							
Very good	1819 (14%)	301 (10%)	272 (14%)	409 (21%)	288 (15%)	392 (20%)	157 (8%)
Good	5058 (39%)	1250 (42%)	699 (35%)	687 (36%)	847 (43%)	639 (32%)	936 (47%)
Moderate	4958 (39%)	1113 (38%)	852 (42%)	748 (39%)	697 (35%)	800 (40%)	748 (37%)
Bad	775 (6%)	228 (8%)	145 (7%)	66 (3%)	70 (4%)	142 (7%)	124 (6%)
Very bad	182 (1%)	43 (1%)	41 (2%)	15 (1%)	17 (1%)	29 (1%)	37 (2%)

### Exploratory factor analysis

After examination of the eigenvalues (eigenvalues: 10.87, 2.44, 1.57, 1.24, 1.13), goodness-of-fit statistics and interpretability of factor structure, the four factor solution was the best solution (χ^2^ = 786.05, df = 227, RMSEA = 0.025; 90%CI = 0.023–0.027, CFI = 0.991) [35]. Nevertheless, a strong major factor (indicated by the high ratio of the first two eigenvalues) was also suggested [17]. There were two items (“household responsibilities difficulty”, “carrying out work and everyday activities difficulty”) that seem to be non-congeneric as they exhibited salient loadings (> 0.40) on two factors [15]; and two others (“immediate recall”, “fold a piece of paper”) that showed similar loadings onto two factors. However, we allowed all items to load on one factor according to their highest loading. In the first factor, items of disabilities of daily living loaded and their loadings ranged from 0.496 to 0.888. The second factor included items of general difficulties in everyday life with loadings ranging from 0.607 to 0.749. The third factor comprised items of impairments and mental health with loadings from 0.310 to 0.670. Finally, factor four was a factor of cognition as cognitive items loaded on this (0.243–0.649) (Table 3).

**Table 3:** Exploratory factor analysis standardised loadings-Parsimax Rotation

Items/Indicators	Factor 1	Factor 2	Factor 3	Factor 4
Household responsibilities difficulty	**0.562**	0.192	0.490	−0.146
Walking a km difficulty	**0.573**	0.050	0.392	−0.033
Washing whole body difficulty	**0.851**	0.003	0.106	0.182
Getting dressed difficulty	**0.888**	0.014	0.048	0.193
Carrying out work & everyday activities difficulty	**0.564**	0.230	0.447	−0.128
Making decisions difficulty	0.057	**0.715**	0.077	0.221
Using the toilet difficulty	**0.496**	0.301	0.109	0.341
Handling money difficulty	0.136	**0.698**	0.062	0.199
Hearing problem	−0.057	0.060	**0.310**	0.084
Eye problem	0.005	0.051	**0.395**	−0.060
Finding right word difficulty	−0.106	**0.749**	0.128	0.100
Change in daily activities	0.037	**0.607**	0.202	0.049
Forgets where he/she is	0.145	**0.644**	0.085	0.290
Difficulty completing chores	0.114	**0.621**	−0.004	0.264
Sleep trouble or recent change in pattern	−0.101	− 0.198	**0.624**	0.050
Feeling of not coping properly with everyday routine	−0.028	0.116	**0.582**	0.043
Gets worn out or exhausted during daytime or evening	−0.229	−0.200	**0.670**	0.122
Time in seconds taken to walk 10 m	**0.410**	0.008	0.030	− 0.063
Learn test	0.074	0.113	0.054	**0.601**
Delayed recall	0.130	0.002	0.072	**0.401**
Long memory test	−0.083	0.062	0.092	**0.642**
Immediate recall	0.118	0.242	0.070	**0.243**
Verbal fluency	0.149	0.126	0.031	**0.556**
Time orientation	0.118	0.146	0.096	**0.649**
Praxis-fold a piece of paper	0.130	0.202	0.038	**0.266**
Story recall difficulty	0.041	−0.002	0.070	**0.617**

### Confirmatory factor analysis

**Fig. 2:**
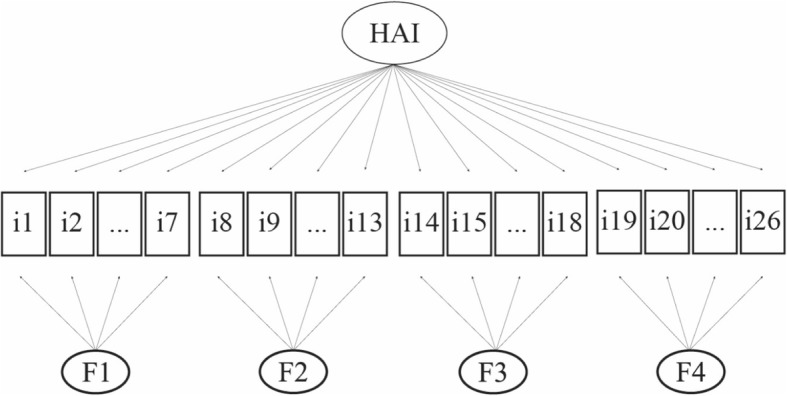
Healthy ageing index bifactor model graphical representation. HAI: Healthy Ageing Index; i1: household responsibilities difficulty; i2: walking a km difficulty; i3: washing whole body difficulty; i4: getting dressed difficulty; i5: carrying out work & everyday activities difficulty; i6: using the toilet difficulty; i7: time in seconds taken to walk 10 m; i8: making decisions difficulty; i9: handling money difficulty; i10: finding right word difficulty; i11: change in daily activities; i12: forgets where he/she is; i13: difficulty completing chores; i14: hearing problem; i15: eye problem; i16: sleep trouble or recent change in pattern; i17: feeling of not coping properly with everyday routine; i18: gets worn out or exhausted during daytime or evening; i19: learn test; i20: delayed recall; i21: long memory test; i22: immediate recall; i23: verbal fluency; i24: time orientation; i25: praxis-fold a piece of paper; i26: story recall difficulty; F1: factor 1; F2: factor 2; F3: factor 3; F4: factor 4

Three different CFA models were tested and compared for the construction of the index; a one-factor model, a second-order model and a bifactor model (Table 4). The one-factor model exhibited acceptable fit based on the CFI (0.902), but had a poorer RMSEA fit (0.073). Hence, the one-factor solution did not seem to be appropriate. Both the second-order factor and the bifactor model exhibited good fit (CFI ≥ 0.90 and RMSEA≤0.06) but the bifactor model exhibited higher CFI and lower RMSEA indexes (Bifactor: CFI = 0.972, RMSEA = 0.041; Second-order: CFI = 0.962, RMSEA = 0.045). In addition, the adjusted chi-square test for model comparison supported as superior the bifactor model as its value was significant when compared to the one-factor (χ^2^ = 5679.77, df = 26, *p* < 0.001) and the second-order model (χ^2^ = 1089.78, df = 22, p < 0.001). Local fit assessment revealed that the bifactor model was the one with the fewest (both in number and in amount) discrepancies between predicted and observed correlations (Additional file 2). As a consequence, for the subsequent analyses the bifactor model was employed Fig. 2. (Additional file 3 presents the item loadings onto the general and the subdomain factors).

**Table 4:** Fit statistics for confirmatory factor analysis models

Model	Chi-square	df	CFI	RMSEA	90%CI	Difftest (chi-square, df)
One factor	14,497.44	299	0.902	0.073	0.072–0.074	(5679.77, 26)^***^
Second-order	5770.29	295	0.962	0.045	0.044–0.046	(1089.78, 22)^***^
Bifactor	4327.95	273	0.972	0.041	0.040–0.042	–

Difftest: an adjusted chi-square difference test; df: degrees of freedom; *RMSEA* Root Mean Square Error of Approximation, *CFI* Comparative Fit Index

^***^ *p* < 0.001

### Measurement invariance

Firstly, we checked if the bifactor model fitted well the empirical data from each country. When we run the model in each country the correlation of the items “washing whole body difficulty” and “getting dressed difficulty” for Puerto Rico was close to 1 (r = 0.989); to avoid multicollinearity only one item was kept to the measurement invariance tests. The bifactor model had acceptable fit in each country (RMSEA values range from 0.030 to 0.052; CFI values range from 0.923–0.976) (Table 5). As acceptable fit was established per country we would expect that configural invariance would also be supported. Goodness-of-fit statistics provided evidence for configural invariance (χ^2^ = 6277.08, df = 1510, RMSEA = 0.046; 90%CI = 0.045–0.047, CFI = 0.958). The hypothesis of scalar invariance (similar loadings and thresholds) was also supported (χ^2^ = 7668.201, df = 1750, RMSEA = 0.047; 90%CI = 0.046–0.049, CFI = 0.948). In addition, the change of CFI and RMSEA was within the predetermined limits (ΔRMSEA = 0.010, ΔCFI = -0.010).

**Table 5:** Model fit for subgroup analyses and for measurement invariance test across countries and gender

Model		Chi-square	df	RMSEA	90%CI	CFI
Country	Cuba	1188.83	260	0.042	0.039–0.044	0.972
	Dominican Republic	1166.40	260	0.050	0.047–0.053	0.924
	Peru	649.06	260	0.033	0.030–0.036	0.976
	Venezuela	1235.53	260	0.052	0.049–0.055	0.923
	Mexico	584.66	260	0.030	0.027–0.033	0.964
	Puerto Rico	1049.99	260	0.046	0.044–0.049	0.971
	Configural	6277.08	1510	0.046	0.045–0.047	0.958
	Scalar	7668.20	1750	0.047	0.046–0.049	0.948
	Difftest^***^	1641.51	240	0.010		−0.010
Gender	Females	3077.07	260	0.040	0.039–0.042	0.964
	Males	1244.39	260	0.031	0.030–0.033	0.975
	Configural	3700.29	502	0.038	0.036–0.039	0.967
	Scalar	4160.82	558	0.038	0.037–0.039	0.963
	Difftest^***^	590.81	56	0.000		−0.004

Difftest: an adjusted chi-square difference test; *df* Degrees of freedom, *RMSEA* Root Mean Square Error of Approximation, *CI* Confidence Intervals, *CFI* Comparative Fit Index

^***^ *p* < 0.001

We also assessed the measurement invariance across men and women separately, applying the same steps as above. Goodness-of-fit statistics supported configural invariance (χ^2^ = 3700.29, df = 502, RMSEA = 0.038; 90%CI = 0.036–0.039, CFI = 0.967) as well as scalar invariance (χ^2^ = 4160.82, df = 558, RMSEA = 0.038; 90%CI = 0.037–0.039, CFI = 0.963). In addition, the change of CFI and RMSEA was within the predetermined limits (ΔRMSEA = 0.000, ΔCFI = -0.004) (Table 5).

### Psychometric coefficients

The general HAI showed excellent reliability (*ω* = 0.96) and based on our bifactor model, *ω*_*H*_ indicated a predominant general factor (*ω*_*H*_ = 0.84). A comparison of *ω*_*H*_ with *ω* (0.84/0.96 = 0.88) showed that most of the reliable variance in total scores could be attributed to the general factor. 12% (0.96–0.84) could be attributed to the multidimensionality caused by the subdomain factors and only 4% was estimated to be random error. Omega hierarchical subscale coefficients were very small (*ω*_*ΗS1*_ = 0.06, *ω*_*ΗS2*_ = 0.02, *ω*_*ΗS3*_ = 0.03, *ω*_*ΗS4*_ = 0.02), showing that little common variance remained after we accounted for the general factor. *ECV* was 0.65 also indicating a quite strong general factor accounting for well over half the common variance; however not exceeding the 0.80 benchmark indicated that part of the variance was also explained by the subdomain factors. *H* value equalled 0.96 indicating that the general factor was a well-defined latent variable [28, 36].

### Concurrent convergent validity

The association between the general factor of healthy ageing and the self-rated health measure, adjusted for age and sex, was significant (standardised estimate = 0.373; bootstrap 95%CI: 0.352–0.394, *p* < 0.001, χ^2^ = 8238.22, df = 348, RMSEA = 0.050; 90%CI = 0.049–0.051, CFI = 0.922) indicating that a one unit increase in the SRH (deterioration of self-rated health) was associated with a 0.373 standardised score increase in the healthy ageing index (higher values indicate worse health).

## Discussion

We showed that a healthy ageing index can be comprised by indicators of intrinsic capacity and functional ability available in different questionnaires. To the best of our knowledge, this the first study creating a healthy ageing index which was tested for various psychometric properties and for measurement invariance. Even though in the literature, there are other successful or healthy ageing indexes [37–39], the novelty of our study lies in the fact that the multifaceted concept of healthy ageing was built by a latent model. Using latent variable modelling to create the healthy ageing index contributes to the creation of a more sensitive measure. Future research on this index will assist in the identification of the most important indicators across the whole range of the latent construct (from the lowest to the highest level of healthy ageing) and of those that are more relevant to people who are in most need. As a consequence, our index will further contribute to person-centered services to the older population.

Regarding the factorial validity of our construct, one-factor model and second-order factor were compared to the bifactor model. We opted for the bifactor structure based on its superior model fit and on its interpretation utility, as there is an ‘inherent statistical bias’ in favour of bifactor models when tested with second-order model [40]. In our study, healthy ageing is conceptualised as the general factor and four subdomain factors, as identified by the EFA. Those subdomain factors are considered common factors as they explain variance above the general construct [41]. A bifactor structure will enable future SEM research to examine the influence of key external covariates both onto the general factor and the subdomain factors; something that is trickier to do in the second-order factor where first- and second-order factors overlap [13].

Measurement invariance, which is fundamental to comparing data among different populations and over time [42] especially when self-reported questionnaires have been employed, was also examined. Our index exhibited excellent measurement invariance properties (configural and scalar invariance) both across ethnic groups and gender, making it possible to meaningfully compare the healthy ageing level of these subpopulations in future research. Furthermore, to assess the concurrent convergent validity of this index with other health measures, we checked its association with the SRH measure adjusted for age and sex to limit any potential moderating effect [43]. The association was moderately strong providing some evidence of our index concurrent convergent validity.

As recommended when a bifactor model is used, we also calculated psychometrically informative bifactor-derived statistics [28, 44]. We calculated *ω* and *ω*_*Η*_ which indicated that a strong percentage of total score variance is attributable to a single general factor. Hence, we can conclude that raw scores can essentially be assumed as indicators of the healthy ageing general factor and are not affected by the multidimensionality of the four subdomain factors. A strong general factor was also indicated from the *ECV* value (*ECV* = 0.65), but as it is less than 0.80 subscale scores should also be considered. However, as the *ω*_*ΗS*_ reliability subscale estimates are low (*ω*_*ΗS1*_ = 0.06, *ω*_*ΗS2*_ = 0.02, *ω*_*ΗS3*_ = 0.03, *ω*_*ΗS4*_ = 0.02) once we account for the general factor, subscale scores have limited added value [45]. We concluded that despite the multidimensionality of the healthy ageing construct, raw scores of the general factor can be interpreted as an essentially unidimensional concept of healthy ageing. Regarding *H* index, its high value (*H* = 0.96) provided support that our general factor is a well-defined latent construct appropriate to be used in future SEM research.

A potential limitation of this study is that only selected catchment urban and rural areas of the countries involved were considered. As a consequence, the generalisability of the findings beyond the specific study sites could have been affected. Moreover, the baseline sample included only people 65 years old and over. Thus, it is possible that our results may not be generalisable to a younger sample.

In addition, in our study we included data from Latin America only, even though 10/66 survey has collected data to catchment areas of China and India as well; the reason behind this decision was that we wanted to initially create a common metric of healthy ageing and examine its properties to a multi-country setting but still culturally and geographically homogeneous sample. In addition, knowing that the Chinese and Indian centres collected data by using English language whereas the Hispanic centres of Latin America used Spanish language also contributed to our selecting of a sub-sample of the whole 10/66 cohort. Future research should focus on the creation of this index to all countries participated in the 10/66 cohort and to the follow-up survey dataset.

Another limitation of this study is that some could argue that our cut-off point considered for factor loadings (±0.20) is not stringent enough [46, 47] or that there were indicators with substantial cross-loadings. As a consequence, these problematic items should not be included in the index. However, for content validity purposes we did not exclude any of the initially employed indicators as all questions seem to be representative and meaningful for measuring health status in an older population [13]. Moreover, to capture various domains of health and create an index representing health status at later years we considered as many indicators as possible from different questionnaires, but with no overlapping [48].

Finally, we assessed the concurrent convergent validity of our index by examining its association with a subjective measurement of health; the self-rated health. Future research should focus on the predictive validity of our index by comparing it with the mortality outcome, which is a more objective measure of an individual’s general health [49] or other indexes related with adverse health outcomes in older people, for instance the frailty index [50].

## Conclusions

There is an emerging need of further empirical work on the scope, construct development and validity of a common healthy ageing metric. Our findings showed that a healthy ageing index with excellent psychometric and measurement invariance properties can be created in a subset of six low-and-middle income countries. As the challenge of global population ageing is constantly growing, especially in low-and-middle income countries [2], replication of our index to other cultural settings and to longitudinal designs will contribute to a more comprehensive understanding of the ageing process. Future research will allow us to validly explore subpopulation differences and key-determinants that could offer new strategies for policy interventions.

## Additional files

Additional file 1 Mplus code.

Additional file 2 Residual differences (differences between predicted and observed correlations) for the one factor, the second-order and the bifactor models to assess local fit assessment.

Additional file 3 Standardised item loadings for the bifactor model.

## Abbreviations

CFA: Confirmatory Factor Analysis
CFI: Comparative Fit Index
CI: Confidence Intervals
df: Degrees of freedom
DRG: Dementia Research Group
ECV: Explained Common Variance
EFA: Exploratory Factor Analysis
HAI: Healthy Ageing Index
ICF: International Classification of Functioning, Disability and Health
MGCFA: Multi-Group Confirmatory Factor Analysis
MIMIC: Multiple-Indicators Multiple-Causes model
RMSEA: Root Mean Square Error of Approximation
SEM: Structural Equation Model
SRH: Self-Rated Health
WHO: World Health Organization
WLSMV: Mean and Variance-adjusted Weighted Least-Squares
*ω*: Omega
*ω*_*H*_: Omega Hierarchical
*ω*_*ΗS*_: Omega Hierarchical Subscales
