# Supplementary material for: Development of a healthy ageing index in Latin American countries - a 10/66 dementia research group population-based study
Source: BMC Med Res Methodol. 2019 Dec 5;19:226. doi: 10.1186/s12874-019-0849-y (PMC6894331; doi:10.1186/s12874-019-0849-y)
Supplement: Supplementary file 1 — Mplus code. [file 12874_2019_849_MOESM1_ESM.docx]

**Additional file 1 (Mplus code)**

!Model for one country (Country 1)

title: Model: Country1

data: file is "Data1.txt";

type is individual;

variable: names are centreid HOUSEID PARTICID gender age countryid Area Region PDAS2 PDAS7 PDAS8 PDAS9 PDAS12 DECIDE TOILET FEED MONEY PPAIN3 PEAR PEYE rswing lswing WORDFIND ACTIV ORIENT CHORES LOSTOUT sleeptrouble routinediff energydiff exhaustion speedtest learntest delayedrecall longmemdiff immedrecalldiff verbalfluency_diff timeorien_diff paper_diff storyrecalldiff PAINFREQ PAINSEV;

usevar are PDAS2 PDAS7 PDAS9 PDAS12 DECIDE TOILET MONEY PEAR PEYE WORDFIND ACTIV ORIENT CHORES sleeptrouble routinediff exhaustion speedtest learntest delayedrecall longmemdiff immedrecalldiff verbalfluency_diff timeorien_diff paper_diff storyrecalldiff;

missing are all (999);

USEOBSERVATION ARE (countryid EQ 1);

categorical are PDAS2 PDAS7 PDAS9 PDAS12 DECIDE TOILET MONEY PEAR PEYE WORDFIND ACTIV ORIENT CHORES sleeptrouble routinediff exhaustion speedtest learntest delayedrecall longmemdiff immedrecalldiff verbalfluency_diff timeorien_diff paper_diff storyrecalldiff;

analysis: Parameterization is Theta;

iterations = 100000;

CONVERGENCE = 0.0005;

model:

GFactor BY

PDAS2* (L1) PDAS7 (L2) PDAS9 (L4) PDAS12@1 (L5) DECIDE (L6) TOILET (L7) MONEY (L8) PEAR (L9) PEYE (L10) WORDFIND (L11) ACTIV (L12) ORIENT (L13) CHORES (L14) sleeptrouble (L15)

routinediff (L16) exhaustion (L17) speedtest (L18) learntest (L19) delayedrecall (L20) longmemdiff (L21) immedrecalldiff (L22) verbalfluency_diff (L23) timeorien_diff (L24) paper_diff (L25) storyrecalldiff (L26);

F1 BY PDAS2* (L11) PDAS7 (L12) PDAS9@1 (L14) PDAS12 (L15) TOILET (L16) speedtest (L17);

F2 BY MONEY* (L21) DECIDE@1 (L22) WORDFIND (L23) ACTIV (L24) ORIENT (L25) CHORES (L26);

F3 BY LEARNTEST@1 (L31) delayedrecall (L32) longmemdiff (L33) immedrecalldiff (L34) verbalfluency_diff (L35) timeorien_diff (L36) paper_diff (L37) storyrecalldiff(L38);

F4 BY PEAR* (L51) PEYE (L52) sleeptrouble (L53) routinediff (L54) exhaustion@1 (L55);

!assumptions of bifactor model.

GFactor WITH F1@0; GFactor WITH F2@0; GFactor WITH F3@0; GFactor WITH F4@0;

f1 with f2@0; f1 with f3@0; f1 with f4@0;

f2 with f4@0;f2 with f3@0;

f3 with f4@0;

! fix residual variance for identification-theta param.

PDAS2@1 PDAS7@1 PDAS9@1 PDAS12@1 DECIDE@1 TOILET@1 MONEY@1 PEAR@1 PEYE@1 WORDFIND@1 ACTIV@1 ORIENT@1 CHORES@1 sleeptrouble@1 routinediff@1 exhaustion@1 speedtest@1 learntest@1 delayedrecall@1 longmemdiff@1 immedrecalldiff@1 verbalfluency_diff@1 timeorien_diff@1 paper_diff@1 storyrecalldiff@1;

output: standardized;

**! Configural model**

title: Model: Configural

data: file is "Data1.txt";

type is individual;

variable: names are centreid HOUSEID PARTICID gender age countryid Area Region PDAS2 PDAS7 PDAS8 PDAS9 PDAS12 DECIDE TOILET FEED MONEY PPAIN3 PEAR PEYE rswing lswing WORDFIND ACTIV ORIENT CHORES LOSTOUT sleeptrouble routinediff energydiff exhaustion speedtest learntest delayedrecall longmemdiff immedrecalldiff verbalfluency_diff timeorien_diff paper_diff storyrecalldiff PAINFREQ PAINSEV;

usevar are countryid PDAS2 PDAS7 PDAS9 PDAS12 DECIDE TOILET MONEY PEAR PEYE WORDFIND ACTIV ORIENT CHORES sleeptrouble routinediff exhaustion speedtest learntest delayedrecall

longmemdiff immedrecalldiff verbalfluency_diff timeorien_diff paper_diff storyrecalldiff;

missing are all (999);

USEOBSERVATIONs ARE (region EQ 1);

grouping is countryid (1=Cuba 2=DominicanRep 3=Peru 4=Venezuela 5=Mexico 20=PuertoRico);

categorical are PDAS2 PDAS7 PDAS9 PDAS12 DECIDE TOILET MONEY PEAR PEYE WORDFIND ACTIV ORIENT CHORES sleeptrouble routinediff exhaustion speedtest learntest delayedrecall longmemdiff immedrecalldiff verbalfluency_diff timeorien_diff paper_diff storyrecalldiff;

analysis: Parameterization is Theta;

ESTIMATOR = WLSMV;

iterations = 1000000;

CONVERGENCE = 0.0009;

model:

GFactor BY PDAS2* !(L1) PDAS7 !(L2) PDAS9 !(L4) PDAS12@1 !(L5) DECIDE !(L6) TOILET !(L7) MONEY !(L8) PEAR !(L9) PEYE !(L10) WORDFIND !(L11) ACTIV !(L12) ORIENT !(L13) CHORES !(L14)

sleeptrouble !(L15) routinediff !(L16) exhaustion !(L17) speedtest !(L18) learntest !(L19) delayedrecall !(L20) longmemdiff !(L21) immedrecalldiff !(L22) verbalfluency_diff !(L23) timeorien_diff !(L24) paper_diff !(L25) storyrecalldiff; !(L26);

F1 BY PDAS2* !(L11) PDAS7 !(L12) PDAS9@1 !(L14) PDAS12 !(L15) TOILET !(L16) speedtest; !(L17);

F2 BY MONEY* !(L21) DECIDE@1 !(L22) WORDFIND !(L23) ACTIV !(L24) ORIENT !(L25) CHORES; !(L26);

F3 BY LEARNTEST@1 !(L31) delayedrecall !(L32) longmemdiff !(L33) immedrecalldiff !(L34) verbalfluency_diff !(L35) timeorien_diff !(L36) paper_diff !(L37) storyrecalldiff;!(L38);

F4 BY PEAR* !(L51) PEYE !(L52) sleeptrouble !(L53) routinediff !(L54) exhaustion@1; !(L55);

!i fix factor means for identification.

[GFactor@0 f1@0 f2@0 f3@0 f4@0];

!assumptions of bifactor model.

GFactor WITH F1@0; GFactor WITH F2@0; GFactor WITH F3@0; GFactor WITH F4@0;

f1 with f2@0; f1 with f3@0; f1 with f4@0;

f2 with f4@0;f2 with f3@0;

f3 with f4@0;

! One threshold in each item are constrained across groups

! One additional threshold in the marker variable are constrained across groups

[PDAS2$1] (t1); [PDAS2$2];

[PDAS7$1] (t3); [PDAS7$2] ;[PDAS7$3] ; [PDAS7$4] ;

[PDAS9$1] (t8); ! marker item

[PDAS12$1] (t9); [PDAS12$2](t10); [PDAS12$3]; !marker item

[DECIDE$1] (t12); ! marker item

[TOILET$1] (t13);

[MONEY$1] (t14);

[PEAR$1] (t15);

[PEYE$1] (t16);

[WORDFIND$1] (t17);

[ACTIV$1] (t18);

[ORIENT$1] (t19);

[CHORES$1] (t20);

[sleeptrouble$1] (t21);

[routinediff$1] (t22);

[exhaustion$1] (t23);

[speedtest$1] (t24); [speedtest$2];

[learntest$1] (t26); [learntest$2](t27); !marker item

[delayedrecall$1] (t28);

[longmemdiff$1] (t29);

[immedrecalldiff$1] (t30);

[verbalfluency_diff$1] (t31); [verbalfluency_diff$2] ;

[timeorien_diff$1](t33);

[paper_diff$1] (t34);

[storyrecalldiff$1] (t35);

! Residual var.

PDAS2@1 PDAS7@1 PDAS9@1 PDAS12@1 DECIDE@1 TOILET@1 MONEY@1 PEAR@1 PEYE@1

WORDFIND@1 ACTIV@1 ORIENT@1 CHORES@1 sleeptrouble@1 routinediff@1 exhaustion@1

speedtest@1 learntest@1 delayedrecall@1 longmemdiff@1 immedrecalldiff@1 verbalfluency_diff@1 timeorien_diff@1 paper_diff@1 storyrecalldiff@1;

!model for the other countries; I provide the code for one (Dominican Republique).

model DominicanRep:

GFactor BY PDAS2* !(L1) PDAS7 !(L2) PDAS9 !(L4) PDAS12@1 !(L5) DECIDE !(L6) TOILET !(L7) MONEY !(L8) PEAR !(L9) PEYE !(L10) WORDFIND !(L11) ACTIV !(L12) ORIENT !(L13) CHORES !(L14)

sleeptrouble !(L15) routinediff !(L16) exhaustion !(L17) speedtest !(L18) learntest !(L19) delayedrecall !(L20) longmemdiff !(L21) immedrecalldiff !(L22) verbalfluency_diff !(L23) timeorien_diff !(L24) paper_diff !(L25) storyrecalldiff; !(L26);

F1 BY PDAS2* !(L11) PDAS7 !(L12) PDAS9@1 !(L14) PDAS12 !(L15) TOILET !(L16) speedtest; !(L17);

F2 BY MONEY* !(L21) DECIDE@1 !(L22) WORDFIND !(L23) ACTIV !(L24) ORIENT !(L25) CHORES; !(L26);

F3 BY LEARNTEST@1 !(L31) delayedrecall !(L32) longmemdiff !(L33) immedrecalldiff !(L34)

verbalfluency_diff !(L35) timeorien_diff !(L36) paper_diff !(L37) storyrecalldiff;!(L38);

F4 BY PEAR* !(L51) PEYE !(L52) sleeptrouble !(L53) routinediff !(L54) exhaustion@1; !(L55);

!assumptions of bifactor model.

GFactor WITH F1@0; GFactor WITH F2@0; GFactor WITH F3@0; GFactor WITH F4@0;

f1 with f2@0; f1 with f3@0; f1 with f4@0;

f2 with f4@0;f2 with f3@0;

f3 with f4@0;

! One threshold in each item are constrained across groups

! One additional threshold in the marker variable are constrained across groups

[PDAS2$1] (t1); [PDAS2$2]; [PDAS7$1] (t3);

[PDAS7$2] ; [PDAS7$3] ; [PDAS7$4] ;

[PDAS9$1] (t8); !marker

[PDAS12$1] (t9); [PDAS12$2](t10); [PDAS12$3]; !marker

[DECIDE$1] (t12);!marker

[TOILET$1] (t13);

[MONEY$1] (t14);

[PEAR$1] (t15);

[PEYE$1] (t16);

[WORDFIND$1] (t17);

[ACTIV$1] (t18);

[ORIENT$1] (t19);

[CHORES$1] (t20);

[sleeptrouble$1] (t21);

[routinediff$1] (t22);

[exhaustion$1] (t23); !marker

[speedtest$1] (t24); [speedtest$2];

[learntest$1] (t26); [learntest$2](t27); !marker

[delayedrecall$1] (t28);

[longmemdiff$1] (t29);

[immedrecalldiff$1] (t30);

[verbalfluency_diff$1] (t31); [verbalfluency_diff$2] ;

[timeorien_diff$1](t33);

[paper_diff$1] (t34);

[storyrecalldiff$1] (t35);

! residual variances

PDAS2*; PDAS7*; PDAS9*; PDAS12*; DECIDE*; TOILET*; MONEY*; PEAR*; PEYE*; WORDFIND*;

ACTIV*; ORIENT*; CHORES*; sleeptrouble*; routinediff*; exhaustion*; speedtest*; learntest*;

delayedrecall*; longmemdiff*; immedrecalldiff*; verbalfluency_diff*; timeorien_diff*; paper_diff*; storyrecalldiff*;

OUTPUT: standardized;

SAVEDATA: DIFFTEST=configural_percountry2.dif;

**!Scalar model**

title: Model: Scalar

data: file is "Data1.txt";

type is individual;

variable: names are centreid HOUSEID PARTICID gender age countryid Area Region PDAS2 PDAS7 PDAS8 PDAS9 PDAS12 DECIDE TOILET FEED MONEY PPAIN3 PEAR PEYE rswing lswing WORDFIND ACTIV ORIENT CHORES LOSTOUT sleeptrouble routinediff energydiff exhaustion speedtest learntest delayedrecall longmemdiff immedrecalldiff verbalfluency_diff timeorien_diff paper_diff storyrecalldiff PAINFREQ PAINSEV;

usevar are countryid PDAS2 PDAS7 PDAS9 PDAS12 DECIDE TOILET MONEY PEAR PEYE WORDFIND ACTIV ORIENT CHORES sleeptrouble routinediff exhaustion speedtest learntest delayedrecall

longmemdiff immedrecalldiff verbalfluency_diff timeorien_diff paper_diff storyrecalldiff;

missing are all (999);

USEOBSERVATIONs ARE (region EQ 1);

grouping is countryid (1=Cuba 2=DominicanRep 3=Peru 4=Venezuela 5=Mexico 20=PuertoRico);

categorical are PDAS2 PDAS7 PDAS9 PDAS12 DECIDE TOILET MONEY PEAR PEYE WORDFIND ACTIV ORIENT CHORES sleeptrouble routinediff exhaustion speedtest learntest delayedrecall longmemdiff immedrecalldiff verbalfluency_diff timeorien_diff paper_diff storyrecalldiff;

analysis: Parameterization is Theta;

DIFFTEST=configural_percountry2.dif; ! scalar vs. configural

ESTIMATOR = WLSMV;

iterations = 100000;

CONVERGENCE = 0.0001;

model:

GFactor BY PDAS2* (L1) PDAS7 (L2) PDAS9 (L4) PDAS12@1 !(L5) DECIDE (L6) TOILET (L7) MONEY (L8) PEAR (L9) PEYE (L10) WORDFIND (L11) ACTIV (L12) ORIENT (L13) CHORES (L14) sleeptrouble (L15) routinediff (L16) exhaustion (L17) speedtest (L18) learntest (L19) delayedrecall (L20) longmemdiff (L21) immedrecalldiff (L22) verbalfluency_diff (L23) timeorien_diff (L24) paper_diff (L25) storyrecalldiff (L26);

F1 BY PDAS2* (L11) PDAS7 (L12) (L13) PDAS9@1 !(L14) PDAS12 (L15) TOILET (L16) speedtest (L17);

F2 BY MONEY* (L21) DECIDE@1 !(L22) WORDFIND (L23) ACTIV (L24) ORIENT (L25) CHORES (L26);

F3 BY LEARNTEST@1 !(L31) delayedrecall (L32) longmemdiff (L33) immedrecalldiff (L34) verbalfluency_diff (L35) timeorien_diff (L36) paper_diff (L37) storyrecalldiff (L38);

F4 BY PEAR* (L51) PEYE (L52) sleeptrouble (L53) routinediff (L54) exhaustion@1; !(L55);

!i fix factor means for identification to the 1st group only.

[GFactor@0]; [f1@0]; [f2@0]; [f3@0]; [f4@0];

!assumptions of bifactor model.

GFactor WITH F1@0; GFactor WITH F2@0; GFactor WITH F3@0; GFactor WITH F4@0;

f1 with f2@0; f1 with f3@0; f1 with f4@0;

f2 with f4@0;f2 with f3@0;

f3 with f4@0;

! fix all thresholds!

[PDAS2$1] (t1); [PDAS2$2] (t2);

[PDAS7$1] (t3); [PDAS7$2] (t4); [PDAS7$3](t5) ; [PDAS7$4](t6) ;

[PDAS9$1] (t8); ! marker item

[PDAS12$1] (t9); [PDAS12$2](t10); [PDAS12$3] (t11); !marker item

[DECIDE$1] (t12); ! marker item

[TOILET$1] (t13);

[MONEY$1] (t14);

[PEAR$1] (t15);

[PEYE$1] (t16);

[WORDFIND$1] (t17);

[ACTIV$1] (t18);

[ORIENT$1] (t19);

[CHORES$1] (t20);

[sleeptrouble$1] (t21);

[routinediff$1] (t22);

[exhaustion$1] (t23);

[speedtest$1] (t24); [speedtest$2] (t25);

[learntest$1] (t26); [learntest$2](t27); !marker item

[delayedrecall$1] (t28);

[longmemdiff$1] (t29);

[immedrecalldiff$1] (t30);

[verbalfluency_diff$1] (t31); [verbalfluency_diff$2] ;

[timeorien_diff$1](t33);

[paper_diff$1] (t34);

[storyrecalldiff$1] (t35);

!residual variances

PDAS2@1 PDAS7@1 PDAS9@1 PDAS12@1 DECIDE@1 TOILET@1 MONEY@1 PEAR@1 PEYE@1 WORDFIND@1 ACTIV@1 ORIENT@1 CHORES@1 sleeptrouble@1 routinediff@1 exhaustion@1

speedtest@1 learntest@1 delayedrecall@1 longmemdiff@1 immedrecalldiff@1 verbalfluency_diff@1 timeorien_diff@1 paper_diff@1 storyrecalldiff@1;

!I provide the code for one of the other countries (Dominican Rep)

model DominicanRep:

GFactor BY PDAS2* (L1) PDAS7 (L2) PDAS9 (L4) PDAS12@1 !(L5) DECIDE (L6) TOILET (L7) MONEY (L8) PEAR (L9) PEYE (L10) WORDFIND (L11) ACTIV (L12) ORIENT (L13) CHORES (L14) sleeptrouble (L15) routinediff (L16) exhaustion (L17) speedtest (L18) learntest (L19) delayedrecall (L20)

longmemdiff (L21) immedrecalldiff (L22) verbalfluency_diff (L23) timeorien_diff (L24) paper_diff (L25) storyrecalldiff (L26);

F1 BY PDAS2* (L11) PDAS7 (L12) PDAS9@1 !(L14) PDAS12 (L15) TOILET (L16) speedtest (L17);

F2 BY MONEY* (L21) DECIDE@1 !(L22) WORDFIND (L23) ACTIV (L24) ORIENT (L25) CHORES (L26);

F3 BY LEARNTEST@1 !(L31) delayedrecall (L32) longmemdiff (L33) immedrecalldiff (L34)

verbalfluency_diff (L35) timeorien_diff (L36) paper_diff (L37) storyrecalldiff (L38);

F4 BY PEAR* (L51) PEYE (L52) sleeptrouble (L53) routinediff (L54) exhaustion@1; !(L55);

!free factor means.

[GFactor*]; [f1*]; [f2*]; [f3*]; [f4*];

!assumptions of bifactor model.

GFactor WITH F1@0; GFactor WITH F2@0; GFactor WITH F3@0; GFactor WITH F4@0;

f1 with f2@0; f1 with f3@0; f1 with f4@0;

f2 with f4@0;f2 with f3@0;

f3 with f4@0;

! fix all thresholds!

[PDAS2$1] (t1); [PDAS2$2] (t2);

[PDAS7$1] (t3); [PDAS7$2] (t4); [PDAS7$3](t5) ; [PDAS7$4](t6) ;

[PDAS9$1] (t8); !marker item

[PDAS12$1] (t9); [PDAS12$2](t10); [PDAS12$3] (t11); !marker item

[DECIDE$1] (t12); !marker item

[TOILET$1] (t13);

[MONEY$1] (t14);

[PEAR$1] (t15);

[PEYE$1] (t16);

[WORDFIND$1] (t17);

[ACTIV$1] (t18);

[ORIENT$1] (t19);

[CHORES$1] (t20);

[sleeptrouble$1] (t21);

[routinediff$1] (t22);

[exhaustion$1] (t23);

[speedtest$1] (t24); [speedtest$2] (t25);

[learntest$1] (t26); [learntest$2](t27); !marker item

[delayedrecall$1] (t28);

[longmemdiff$1] (t29);

[immedrecalldiff$1] (t30);

[verbalfluency_diff$1] (t31); [verbalfluency_diff$2] ;

[timeorien_diff$1](t33);

[paper_diff$1] (t34);

[storyrecalldiff$1] (t35);

! Residual variances

PDAS2*; PDAS7*; PDAS9*; PDAS12*; DECIDE*; TOILET*; MONEY*; PEAR*; PEYE*; WORDFIND*;

ACTIV*; ORIENT*; CHORES*; sleeptrouble*; routinediff*; exhaustion*; speedtest*; learntest*;

delayedrecall*; longmemdiff*; immedrecalldiff*; verbalfluency_diff*; timeorien_diff*; paper_diff*; storyrecalldiff*;

output: standardized;tech5;
