# Supplementary material for: Development of a healthy ageing index in Latin American countries - a 10/66 dementia research group population-based study
Source: BMC Med Res Methodol. 2019 Dec 5;19:226. doi: 10.1186/s12874-019-0849-y (PMC6894331; doi:10.1186/s12874-019-0849-y)
Supplement: Supplementary file 3 — Standardised item loadings for the bifactor model. [file 12874_2019_849_MOESM3_ESM.docx]

**Additional file 3 (Standardised Item loadings-bifactor model)**

| Items | Items/Indicators Label | General Factor | Factor1 | Factor2 | Factor3 | Factor4 |
| --- | --- | --- | --- | --- | --- | --- |
| PDAS2 | household responsibilities difficulty | 0.607 | 0.695 |  |  |  |
| PDAS7 | walking a km difficulty | 0.556 | 0.612 |  |  |  |
| PDAS8 | washing whole body difficulty | 0.771 | 0.604 |  |  |  |
| PDAS9 | getting dressed difficulty | 0.763 | 0.590 |  |  |  |
| PDAS12 | carrying out work & everyday activities difficulty | 0.654 | 0.649 |  |  |  |
| DECIDE | making decisions difficulty | 0.806 |  | 0.409 |  |  |
| TOILET | using the toilet difficulty | 0.903 | 0.209 |  |  |  |
| MONEY | handling money difficulty | 0.852 |  | 0.327 |  |  |
| PEAR | hearing problem | 0.297 |  |  | 0.343 |  |
| PEYE | eye problem | 0.267 |  |  | 0.433 |  |
| WORDFIND | finding right word difficulty | 0.657 |  | 0.423 |  |  |
| ACTIV | change in daily activities | 0.678 |  | 0.264 |  |  |
| ORIENT | forgets where he/she is | 0.877 |  | 0.212 |  |  |
| CHORES | difficulty completing chores | 0.727 |  | 0.346 |  |  |
| Q541 | sleep trouble or recent change in pattern | 0.151 |  |  | 0.546 |  |
| Q643 | feeling of not coping properly with everyday routine | 0.492 |  |  | 0.429 |  |
| Q481 | gets worn out or exhausted during daytime or evening | 0.171 |  |  | 0.615 |  |
| NEO12B | time in seconds taken to walk 10 metres | 0.247 | 0.289 |  |  |  |
| LEARN | learn test | 0.540 |  |  |  | 0.467 |
| WORDDEL | delayed recall | 0.382 |  |  |  | 0.308 |
| LONGMEM | long memory test | 0.476 |  |  |  | 0.499 |
| WORDIMM | immediate recall | 0.552 |  |  |  | 0.066 |
| ANIMALS | verbal fluency | 0.615 |  |  |  | 0.380 |
| TIMEORIENT | time orientation | 0.690 |  |  |  | 0.429 |
| PAPER | praxis-fold a piece of paper | 0.475 |  |  |  | 0.150 |
| STORY | story recall difficulty | 0.538 |  |  |  | 0.451 |
